# Acquired Von Willebrand Syndrome as a Presenting Manifestation of Monoclonal IgM-Producing Non-Hodgkin’s B-cell Lymphoma

**DOI:** 10.7759/cureus.73867

**Published:** 2024-11-17

**Authors:** Nur Rahman, Aaron Lobo, Amy Gates

**Affiliations:** 1 Internal Medicine, Bridgeport Hospital/Yale New Haven Health, Bridgeport, USA; 2 Hematology/Oncology, Bridgeport Hospital/Yale New Haven Health, Bridgeport, USA

**Keywords:** acquired von willebrand syndrome, bleeding disorders, non-hodgkin's lymphoma, pre-op planning, waldenstrom's macroglobulinemia

## Abstract

Acquired von Willebrand syndrome (aVWS) is a rare hematological disorder depicted by dysfunctional or deficient von Willebrand factor activity and can be associated with underlying hematological malignancies such as non-Hodgkins lymphoma (NHL). This case report examines a patient with aVWS secondary to NHL and highlights the challenges in managing bleeding and optimizing a patient for surgery. The case report will explore the multidisciplinary approach to recognizing aVWS, treatment modalities, and adjunctive measures to reduce bleeding risk as well as long-term management.

## Introduction

Acquired von Willebrand syndrome (aVWS) is a rare bleeding disorder distinguished by abnormalities in the quality, function, or structure of von Willebrand factor (VWF) [1]. It is characterized by laboratory findings akin to those seen in inherited von Willebrand disease but differs in the absence of a bleeding history in the individual or their family [1,2]. It was initially recognized over 50 years ago when it was documented in a patient with systemic lupus erythematosus. Since then, aVWS has been described in association with various conditions such as lymphoproliferative disorders, myeloproliferative neoplasms, solid organ tumors, autoimmune disorders, and cardiovascular disease [3,4]. We describe a case of non-Hodgkin’s lymphoma with IgM monoclonal gammopathy that presented with acquired von Willebrand syndrome. We will examine the proposed mechanisms of this entity as well as presentation, clinical course, and treatment of aVWS in the setting of hematological malignancy.

## Case presentation

**Figure 1:**
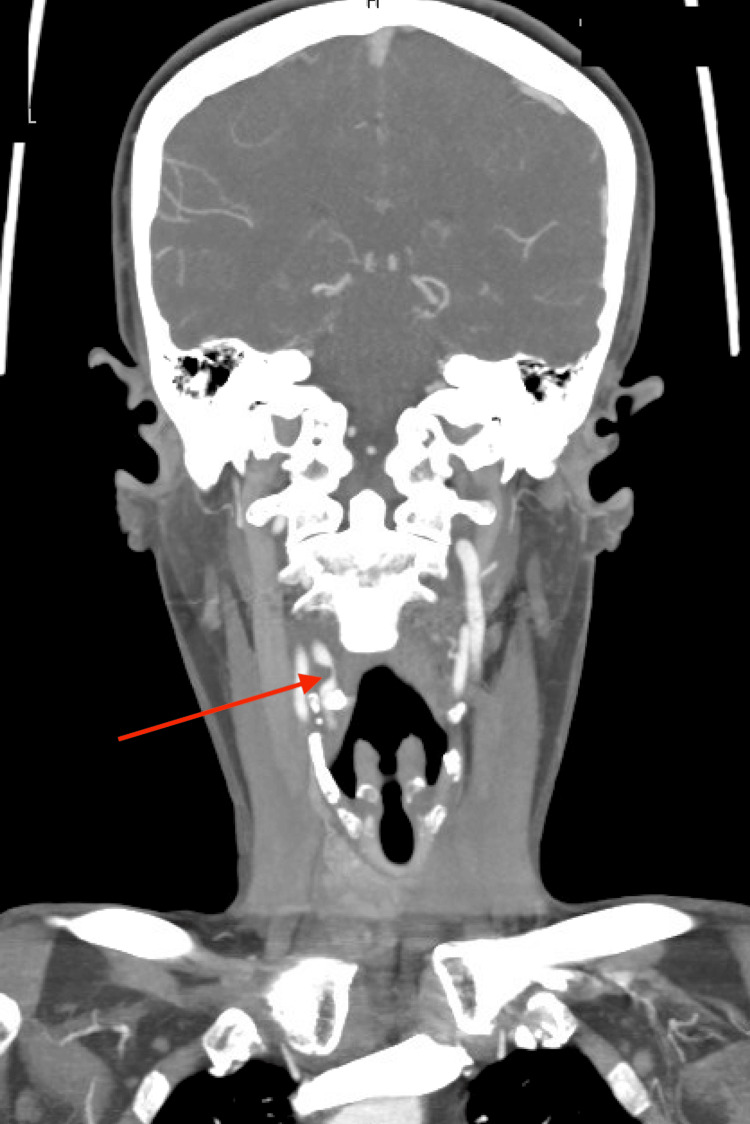
Computer Tomography Angiogram of Head and Neck Showing Critical Stenosis of Proximal Internal Carotid Artery (Red Arrow)

A 60-year-old female with a past history of Hodgkin’s lymphoma treated 30 years prior with lymph node resection, radiation, and staging splenectomy presented with increasing occipital headaches, blurred vision, light-headedness, and syncope. Her medical history included several prior surgeries: hemithyroidectomy, left geniculate neuralgia status post (s/p) surgery complicated by abscess requiring three separate craniotomies, and splenectomy without any perioperative bleeding complications. She had noticed easy bruising for the month prior to her presentation, weight loss of 15 pounds over six months, and easy excessive bleeding with minor trauma. She denied any mucosal bleeding, epistaxis, or spontaneous hematomas. Imaging with computerized tomography (CT) of the head and CT angiography of the head and neck revealed critical stenosis of the right proximal carotid artery stenosis requiring an endarterectomy (Figure 1).

She was initiated on aspirin and clopidogrel but noted worsening bruising. Antiplatelet therapy was discontinued. Concern for a bleeding diathesis prompted a preoperative workup that showed a normal platelet count of 444,000 (cells/mm^3^), normal prothrombin time (9.8 sec), normal partial thromboplastin time (30.1 sec), and normal fibrinogen (486 mg/dL). Further evaluation revealed a low factor VIII (FVIII) level (32.8%), low von Willebrand factor antigen (VWF:Ag) (29%), and reduced activity (VWF:RCo) (<19%) (Table 1). Multimeric analysis was abnormal and revealed the absence of the largest multimers. A low VWF:RCo/Ag ratio (<0.7), abnormal multimeric analysis, and the absence of a past or family history of bleeding strongly suggested bleeding due to aVWS. Preoperatively, a trial of antihemophilic factor/VWF complex and intravenous immunoglobulin improved both VWF and FVIII levels significantly. These levels were further monitored post-administration of replacement therapy to determine optimum dosing and frequency prior to her surgery. She successfully underwent carotid endarterectomy with the above regimen without bleeding complications. Postoperatively, antiplatelet therapy was withheld given concern for bleeding.

**Table 1:** Coagulation Panel and Von Willebrand Factor Levels and Activity

Laboratory Data	Patients Initial Values	Patients Values Post-Treatment
Prothrombin time	9.8 sec	10.0 sec
Partial thromboplastin time	30.1 sec	32.2 sec
Factor VIII (FVIII) levels	32.8% IU/ml	85.8% IU/ml
von Willebrand factor antigen (VWF:Ag)	29%	68%
von Willebrand activity (VWF:RCo)	<19%	26%

Further evaluation of her normocytic anemia that had been noted at presentation included a paraprotein evaluation which showed a monoclonal gammopathy that was determined to be of IgM-lambda (0.12 g/dL) sub-type on serum immunofixation. Immunoglobulin quantification revealed an increase in IgM levels (284 mg/dL; normal: 40-230 mg/dL) with normal free kappa/lambda chain ratio (Table 1). Peripheral blood flow cytometry revealed a population of monoclonal cells (9%-10%) with CD19dim+, CD20dim+, CD5dim+, CD23-, CD10-, CD103-, CD11c-, FMC7+, CD38-, CD43-, IgG-, IgMdim/lambda chain restricted, and cyclin D1 negative concerning for monoclonal B-cell lymphoproliferative disease. CT imaging of the chest and abdomen revealed asplenia and no lymphadenopathy. Rheumatologic evaluation included antinuclear antibody and rheumatoid factor which were both negative. Given concern for a hematological malignancy, she underwent a bone marrow evaluation with flow cytometry findings identical to peripheral blood flow cytometry. Bone marrow biopsy showed a mature trilineage hematopoiesis. CyclinD1 was negative. A diagnosis of small lymphocytic lymphoma (low-grade B-cell non-Hodgkin’s lymphoma) with IgM gammopathy was made. Staging positron emission tomography (PET) scan was completed that did not reveal evidence of gross pathologic by size adenopathy in the neck, chest, abdomen, or pelvis. She was initiated on bendamustine and rituximab with the intent of treating the underlying lymphoma and consequently her aVWS.

## Discussion

Monomeric von Willebrand factor (VWF) facilitates hemostasis by forming high molecular weight (HMW) multimers that form a scaffolding which allows platelet binding to vessel wall collagen [5]. Inherited Von Willebrand disease (VWD) is characterized by either a net decrease in VWF monomers (as seen in Type 1 and Type 3) or abnormalities in HMW multimer formation (as seen in Type 2). Acquired von Willebrand syndrome (aVWS) can occur due to a variety of pathogenic mechanisms, the majority of which contribute to an elevated degradation or clearance of circulating von Willebrand factor [2]. Antibodies bound to VWF, produced either by autoimmune disorders or clonal plasma cells, alter VWF, leading to an increased clearance of VWF by the reticuloendothelial system [6]. Alternatively, antibodies can interfere with the function of VWF and affect its binding with platelets (at glycoproteins Ib and IIb/IIIa receptor sites) and collagen [7,8]. Adsorption of HMW multimers onto myeloma cells and onto platelets in thrombocytosis has also been noted. Proteolytic cleavage of HMW multimers also results in bleeding [9]. This occurs due to shear stress-induced unfolding and has been demonstrated in severe aortic stenosis and in patients with left ventricular assist devices [10].

This case was characterized by an IgM monoclonal gammopathy in the setting of non-Hodgkin B-cell lymphoproliferative disorder. The mechanisms of AVWS may differ between IgG and IgM gammopathy. Loss of HMW multimers has been specifically noted in the presence of an IgM gammopathy and may affect the treatment options [11]. Classically Waldenstrom’s macroglobulinemia is associated with elevated IgM levels and can be complicated by hyperviscosity. High serum viscosity has been noted to increase shear forces in circulating blood that may contribute to HMW breakdown [12].

A diagnosis of aVWS is considered in an older individual without a personal or family history of bleeding who presents with moderate mucocutaneous bleeding [13]. Bleeding can be severe in the setting of trauma or following surgery especially in patients with low factor VIII antigen levels [1]. The International Society on Thrombosis and Haemostasis has noted that aVWS associated with lymphoproliferative disorders may have more severe bleeding manifestations compared to other etiologies [14]. Preliminary laboratory investigations should include platelet count, prothrombin time (PT), and a partial thromboplastin time (PTT). A prolonged PTT may be the first indicator of a VWF abnormality if it is associated with diminished factor VIII levels. Additional workup should include von Willebrand factor antigen (VWF:Ag), von Willebrand factor ristocetin cofactor activity (VWF:RCo), activity/antigen ratio (VWF:Ag/VWF:RCo), and HMW multimeric analysis help confirm the diagnosis [15]. VWF:RCo/Ag ratio reduction in aVWS can indicate the presence of an inhibitory antibody or a selective loss of HMW multimers which was noted in this case. HMW multimeric analysis confirmed the loss of large HMW multimers.

Identifying the underlying etiology of aVWS helps to optimize treatment. Treatment of aVWS is usually directed at controlling acute bleeding events and maintaining long-term hemostasis. Treatment of the underlying etiology is the only option for cure [1,2].

Therapies for acute bleeding are directed toward increasing VWF levels and reducing the effect of inhibitors. Desmopressin (DDVAP) can be administered intravenously or subcutaneously and has been shown to increase VWF levels transiently and is especially useful in patients undergoing invasive or surgical procedures. The response to DDVAP varies depending on the underlying etiology of aVWS with an overall response rate of 30% [14]. The best response rates have been noted in patients with underlying lymphoproliferative disorders (44%) [14]. Factor replacement therapy using plasma-derived concentrates containing VWF can be used in aVWS but with shortened response times. Close monitoring antigen and VWF:RCo/VWF:CB levels and tailoring of therapy are necessary. Antifibrinolytic therapy, such as ε-aminocaproic acid, can be used as an adjunct to DDVAP and factor replacement especially for patients undergoing surgeries that are high risk for bleeding such as gastrointestinal tract and oral cavity. Plasmapheresis and intravenous immunoglobulins (IVIG) have also been used in the setting of autoantibody-driven VWF dysfunction. IVIG has been noted to be more effective for IgG gammopathies (either autoimmune or clonal) [11,14]. The mechanism of action of IVIG is comparable to its role in acquired FVIII deficiency in that the anti-idiotype antibodies contained in the Ig preparation interfere with autoantibodies produced. Alternative hypotheses include blockade of Fc-receptors on reticulo-endothelial complexes that clear auto-antibody bound VWF. Plasmapheresis is more effective at reducing IgM levels and improving blood viscosity [16].

This case illustrated the challenge of managing the thrombotic risk in patients with pre-existing cardiovascular disease in patients with aVWS. Interestingly, the role of VWF levels as a surrogate of endothelial integrity and function has been studied to determine its role as a cardiovascular risk factor [17]. Epidemiological and in-vitro data obtained point toward a possible albeit weak association of elevated VWF levels and increased adverse outcomes like stroke and myocardial infarction especially in high-risk individuals [17]. Although there are no formal guidelines, general recommendations have been to avoid antiplatelet and anticoagulant use in patients with aVWS if feasible. We avoided the use of antiplatelets both pre- and postoperatively [2]. The cause for aVWS in this case was likely the underlying lymphoproliferative disorder (LPD) which was treated with bendamustine and rituximab regimen. The degree of response of aVWS in patients with Waldenstrom's macroglobulinemia (WM) has been shown to be related to the response of WM to disease-specific treatment [18].

## Conclusions

In conclusion, this case report describes a patient with a rare presentation of acquired von Willebrand’s syndrome who was diagnosed with IgM gammopathy due to non-Hodgkin’s lymphoproliferative disorder. The treatment of aVWS is twofold: managing the acute bleed with factor replacement/desmopressin while initiating treatment of the underlying etiology in an effort to achieve remission.
